# Antibiotic and anti-inflammatory use and the risk of prostate cancer

**DOI:** 10.1186/1756-0500-2-57

**Published:** 2009-04-17

**Authors:** Nicholas A Daniels, Yea-Hung Chen, Stephen Bent

**Affiliations:** 1 University of California, San Francisco, Department of Medicine, Division of General Internal Medicine, San Francisco, California, USA; 2 Veteran Affairs Medical Center, San Francisco, California, USA; 3 University of California, San Francisco, Department of Medicine, Division of General Internal Medicine, Veteran Affairs Medical Center, San Francisco, California, USA

## Abstract

**Background:**

Prostate inflammation or infection may increase the risk of prostate cancer. Antibiotics and non-steroidal anti-inflammatory drugs (NSAIDs) are used to treat prostatitis and urinary tract infections (UTIs). The objective of our study was to assess whether their use decreases the risk of prostate cancer.

**Methods:**

We conducted a case-control study among men with incident prostate cancer (N = 65 cases) and without prostate cancer (N = 195 controls) at the San Francisco Veteran Affairs medical center (VAMC) between June 1996 and June 2006. Cases were all patients who had prostate biopsies positive for cancer. We matched controls to cases on age group and race at a 3:1 ratio, and each matched pair was given an identical index date. Total antibiotic, aspirin, and NSAID use (number of prescriptions) was computed for each participant by drug type and was restricted to a fill date at least 1 year before the index date. Logistic regression was used for analysis. We adjusted for the matching variables (age group and race) and potential confounders (years of VAMC enrollment and number of clinic visits).

**Results:**

Neither total antibiotic use nor total anti-inflammatory use reduces the risk of prostate cancer (*P *> 0.05).

**Conclusion:**

Our analysis did not reveal a relation between use of antibiotics, aspirin, or NSAIDs and the risk of prostate cancer.

## Background

Prostate cancer is a major cause of morbidity and mortality in the United States and worldwide. Age, race, and family history are known risk factors for prostate cancer, but there is also limited biological and epidemiological evidence that suggest prostate inflammation or infection, also known as prostatitis, may increase the risk of prostate cancer [1,2]. Antibiotics and non-steroidal anti-inflammatory drugs (NSAIDs) are often used to treat prostatitis and urinary tract infections (UTIs) in men. Although prostatitis may be present in patients diagnosed with prostate cancer, the prevalence and incidence of prostatitis are thought to exceed that of prostate cancer [1-6].

Our hypothesis is that antibiotic use and/or use of NSAIDs may decrease the risk of prostate cancer. There is strong and consistent evidence from animal and laboratory studies, which suggest that regular use of NSAIDs may reduce prostate cancer risk [7-9]. Previous studies also indicate that NSAIDs have an inhibitory effect on prostate cancer cells, which suggests that prostaglandins play a pivotal role in prostate cancer biology [10-15]. Although cyclooxygenase-mediated production of prostaglandins appears to play an important role in the biology of prostate cancer, NSAIDs may have several mechanisms of action against prostate cancer, including apoptosis, inhibition of angiogenesis and cellular growth [16,7-9].

Chemoprevention of prostate cancer, which is the primary focus of our study, evaluates drugs which may reduce the risk of prostate cancer with the goal of reducing the incidence of prostate cancer, as well as reducing treatment-related morbidity [17]. Our study examines whether known treatment for prostatitis, such as antibiotics and anti-inflammatory drugs, decreases the risk of subsequent prostate cancer. This is the first study to evaluate the effect of antibiotics on prostate cancer risk.

## Methods

To investigate our hypothesis that antibiotics, aspirin, and NSAIDs (refers only to non-aspirin, nonselective NSAIDs) decrease the risk of incident prostate cancer, we conducted a case-control study of patients diagnosed with prostate cancer and compared them to general internal medicine clinic-based controls without known prostate cancer, frequency-matched to cases on age and race/ethnicity. Our study design is similar to studies performed evaluating the association between antibiotics and breast cancer [18-20].

We used computerized medical record information from the San Francisco VAMC. Patients eligible for the study were men enrolled at SF Veterans Administration Medical Center (VAMC) system before July 1, 2000 and were at least 40 years of age or older at the time of VAMC enrollment. In addition, patients had to have at least one prostate specific antigen (PSA) test in the past 10 years (between June 1996 and June 2006) and must have been seen in a General Medicine Practice Clinic on two or more occasions between June 1996 and June 2006. The study protocol was approved by the Committee on Human Research of the University of California, San Francisco. Variables extracted included race and ethnicity, prostate biopsy results, prostate cancer diagnosis, history of acute or chronic prostatitis; number of health care visits, history of UTIs (clinically diagnosed or urine testing with white blood cell count of >10), history of benign prostatic hyperplasia (BPH). The pharmacy database was used to determine the amount and duration of antimicrobial and non-steroidal anti-inflammatory use (including the cumulative number of days of medication use and the total number of prescriptions) for the following medications: antibiotics (macrolides, azithromycin, erythromycin, clarithromycin, tetracyclines, doxycycline, penicillins, cephelexin, cephalosporins, sulfonamides, TMP-SMX, ciprofloxacin, levofloxacin), antivirals, antifungals, anti-inflammatory medications (non-steroidal anti-inflammatory medication, COX-2 inhibitors, aspirin, anti-TNF medications), and other medications of interest (testosterone, finasteride, alpha receptor blockers).

We identified 65 patients with a recorded biopsy-positive prostate cancer. We conducted a case-control study among men with incident prostate cancer (N = 65 cases) and without prostate cancer (N = 195 controls) at the SF VAMC between June 1996 and June 2006. Cases were all patients who had prostate biopsies positive for cancer. The prostate cancer diagnosis date was designated as the index date. We randomly matched controls to cases on age group and race at a 3:1 ratio, and each matched pair was given an identical index date. Total antibiotic (anti-bacterial agents only), antimicrobial use (antibacterial, antivirals, and antifungals combined) and NSAIDs, or aspirin use (number of prescriptions) was computed for each participant by drug type and was restricted to a fill date at least 1 year before the index date. Logistic regression was used for analysis. We adjusted for the matching variables (age group and race, which are well-accepted risk factors for prostate cancer) as well as for potential confounders (years of VAMC enrollment and number of clinic visits) which increase the likelihood of having a prostate cancer diagnosis. We used STATA 9.2 (STATCORP, College Station, TX) for statistical analysis. A multiple logistic regression model was used to model the overall association of antibiotics, antimicrobials, NSAIDs, aspirin use and multiple covariates and was used to calculate the odds ratios (ORs) and 95% confidence intervals (CIs). Quantity of medication use was grouped into five categories according to increasing number of prescriptions prescribed.

## Results

Demographic and some health characteristics for the VA sample are reported in Table 1. In our analysis, 65 case-patients and 195 control-patients were included in our analysis. Median age of case-patients was 78 years (range: 51–99 years) vs. 77 years (range: 52–96 years) for control-patients. The majority of study participants were White (43%) or Black (26%). Although not statistically significant, case-patients (87%) were more likely than control-patients (77%), to have a history of UTI (*P *= .08). Case-patients had a greater number of clinic visits (mean 146) compared to control-patients (mean 123) *P *= 0.01. There were 14 (7%) control-patients who had finasteride use, with an average of 398.6 doses per individual. None of the prostate cancer patients had prior finasteride use.

**Table 1:** Characteristics of VA Men Cases and Controls

	Cases (n = 65)	Controls (n = 195)	*p *value †
Current age			
≤ 55	2 (3.1%)*	6 (3.1%)	0.98
55–65	7 (10.8%)	21 (10.8%)	
65–75	17 (26.2%)	51 (26.2%)	
75–85	27 (41.5%)	81 (41.5%)	
85–95	11 (16.9%)	33 (16.9%)	
> 95	1 (1.5%)	3 (1.5%)	

Race			
Asian or Pacific Islander	3 (4.6%)	9 (4.6%)	1
Black	17 (26.2%)	51 (26.2%)	
Latino or Hispanic	2 (3.1%)	6 (3.1%)	
White	28 (43.1%)	84 (43.1%)	
Unknown	15 (23.1%)	45 (23.1%)	

Number of visits			
≤ 50	7 (10.8%)	44 (22.6%)	0.01
50–100	18 (27.7%)	63 (32.3%)	
100–150	20 (30.8%)	48 (24.6%)	
> 150	20 (30.8%)	40 (20.5%)	

Years of enrollment			
≤ 10	12 (18.5%)	23 (11.8%)	0.25
10–15	16 (24.6%)	55 (28.2%)	
15–20	11 (16.9%)	46 (23.6%)	
> 20	26 (40.0%)	71 (36.4%)	

History of prostatitis	1 (1.54%)	8 (4.1%)	0.46

History of BPH	41 (63.1%)	114 (58.5%)	0.56

Elevated PSA (>4.0)	21 (32.3%)	27 (13.9%)	<0.05

History of UTI	57 (87.7%)	151 (77.4%)	0.08

† The comparisons for current age, number of visits, and year of enrollment use the Wilcoxon rank sum test; all other comparisons use Fisher's exact test.

* n (%), number in parentheses are a percentage.

In a multiple logistic regression model (Table 2), after adjustment for the matching variables (age group and race), as well as the potential confounders (number of UTIs, years of enrollment, and number of visits), total antibiotic use, antimicrobial use, anti-inflammatory use, or aspirin use did not reduce the risk of incident prostate cancer. A higher levels of antibiotic, NSAIDs, and aspirin use was also not associated with a reduced risk of prostate cancer (tests for trend, p = 0.33, 0.23, and 0.35, respectively). Subgroup analysis for specific classes of antibiotics (cephalosporins, macrolides, penicillins, quinolones, sulfonamides, and tetracyclines) also did not reveal any significant effect on prostate cancer risk (data not shown).

**Table 2:** Antibiotic and Anti-Inflammatory Use and Prostate Cancer Risk: Results from a Logistic Regression Model

**No. antibiotic prescriptions**	**OR (95% CI)**	**Trend *P-value***
0	Reference	0.33

1–25	1.31 (0.50,3.40)	

26–50	0.60 (0.20,1.82)	

51–100	0.62 (0.22,1.76)	

101^+^	0.78 (0.30,1.98)	

**No. anti-inflammatory prescriptions**		

0	Reference	0.25

1–500	0.88 (0.41,1.91)	

501–1000	0.28 (0.07,1.15)	

1001–1500	0.62 (0.20,1.90)	

1501^+^	0.62 (0.24,1.59)	

**No. aspirin prescriptions**		

0	Reference	0.35

1–500	0.60 (0.26,1.38)	

501–1000	0.43 (0.13,1.42)	

1001–1500	0.40 (0.12,1.38)	

1501^+^	1.04 (0.36,3.00)	

*ORs adjusted for age group, race, years of enrollment, and number of visits

## Discussion

Our analysis did not reveal an association between the use of antibiotics, NSAIDs, or aspirin and the risk of prostate cancer. Our study is the first report to assess the relationship between prostate cancer and antibiotics. It is plausible that some classes of antibiotics may have an effect on prostate cancer by reducing prostate infections and inflammatory responses. Both microbiological and epidemiologic studies show that microbes that commonly infect or inflame the prostate may be associated with prostate cancer [21-28]. To further clarify whether antimicrobial agents influence prostate cancer, studies in a larger sample, given our wide confidence intervals, should be strongly considered to definitively answer this research question. For example, the confidence interval examining the effect of antibiotics (0.30 to 1.98) includes a potential 70% reduction in the odds of prostate cancer among the highest users of antibiotics.

Previous epidemiological studies have assessed the association of NSAIDs with prostate cancer. Studies have found that NSAIDs or aspirin may reduce the risk of prostate cancer; differences have been found between NSAIDs and aspirin use and prostate cancer risk, [10-13] so our evaluation also analyzed these classes of medications separately. In a cross-sectional, self-administered survey of over 1200 Canadian men, aspirin use was associated with a 42% reduction in the odds of prostate cancer, but NSAIDs were not related to prostate cancer risk [12]. In a longitudinal study of predominantly white men in Minnesota (n = 1362), age 50–79 years, NSAIDs use had a relative odds of 0.45 (95% CI 0.28–0.73) with 6 years of follow-up [13]. Unfortunately, this study only assessed NSAIDs use at baseline [13]. In a case-control study of men age 65 years and older who had undergone prostate biopsy, NSAIDs/COX-2 inhibitors or aspirin use during a 2 year period reduced the likelihood of prostate cancer with over 2000 cases and similar number control participants with NSAIDs/COX-2 inhibitors: OR 0.71 (95% CI 058–0.86) and aspirin: OR 0.84 (95% CI 0.74–0.96) [11]. A systematic review of 12 studies (5 retrospective and 7 prospective) found a summary odds ratio for aspirin and prostate cancer risk of 0.9 (95% CI 0.82–0.99), but reduction in risk with the use of other NSAIDs was less consistent with a combined OR 0.87 (95% CI 0.61–1.23) [10]. Despite substantial evidence of a reduced incidence of prostate cancer with NSAIDs or aspirin use, neither medication is currently prescribed as a primary chemopreventative measure for prostate cancer, given potential medication side effects and the lack of data from a randomized controlled trial.

Potential limitations of our study should be recognized. We limited our analysis to the number of pills prescribed to patients and filled by patients. It is unknown whether patients actually took the medications that were prescribed once their prescriptions were filled. Thus, the exposure measure may have incorrectly categorized patients according to the number of medication exposures. In addition, VA patients often see physicians outside of the VA system and may have obtained prescriptions outside of the VA or used over-the-counter NSAIDs, which we could not account for in our analysis. Another potential limitation is that our study sample mean age was 78, which is somewhat older than the average age of 70 at time of diagnosis for prostate cancer [29]. This study was not able to assess whether antibiotics and anti-inflammatories have a different risk in younger patients. Future studies assessing the relationship between antibiotic use and prostate cancer risk should consider including only men who are at particularly high risk for prostate cancer, such as those with high-grade prostatic intraepithelial neoplasia (HG-PIN) on prostate biopsies (a known precursor of prostate cancer) or those with strong family histories or African American race, instead of selecting all men which may mitigate detection of true effect. Limiting the selection of patients to those who have undergone a prostate biopsy may also reduce, but not eliminate, the possibility of detection bias.

## Conclusion

In summary, current data from the San Francisco VAMC database does not provide evidence of support for the hypothesis that antibiotics or NSAIDs decrease the risk of prostate cancer.

## Competing interests

The authors declare that they have no competing interests.

## Authors' contributions

All authors contributed to study conduct and design. ND oversaw study design. ND, YH-C and SB acquired data. YH-C conducted statistical analysis. All authors interpreted data. ND prepared and revised manuscript. All authors read and approved the final manuscript.

## References

[B1] Roberts RO, Bergstralh EJ, Bass SE, Lieber MM, Jacobsen SJ (2004). Prostatitis as a risk factor for prostate cancer. Epidemiology.

[B2] Dennis LK, Lynch CF, Torner JC (2002). Epidemiologic association between prostatitis and prostate cancer. Urology.

[B3] Roberts RO, Jacobson DJ, Girman CJ, Rhodes T, Lieber MM, Jacobsen SJ (2002). Prevalence of prostate-like symptoms in a community based cohort of older men. J Urology.

[B4] McNaughton M, Meigs JB, Barry MJ, Walker Corkey E, Giovannucci E, Kwachi I (2002). Prevalence and correlates of prostatitis in the health professionals follow-up study cohort. J Urology.

[B5] Weidner W, Schiefer HG, Krauss H, Jantos C, Friedrich HJ, Altmannsberger M (1991). Chronic prostatitis: a thorough search for etiologically involved microorganisms in 1,461 patients. Infection.

[B6] Collins MM, Stafford RS, O'Leary MP, Barry MJ (1998). How common is prostatitis? A National Survey of Physician Visits. J Urology.

[B7] Kirschenbaum A, Liu X, Yao S, Levine AC (2001). The role of cyclooxygenase-2 in prostate cancer. Urology.

[B8] Kirschenbaum A, Klausner AP, Lee R, Unger P, Yao S, Liu XH, Levine AC (2000). Expression of cyclooxygenase-1 and cyclooxygenase-2 in the human prostate. Urology.

[B9] Liu XH, Kirschenbaum A, Yao S, Lee R, Holland JF, Levine AC (2000). Inhibition of cyclooxygenase-2 suppresses angiogenesis and the growth of prostate cancer in vivo. J Urol.

[B10] Mahmud S, Franco E, Aprikian A (2004). Prostate cancer and use of nonsteroidal anti-inflammatory drugs: systematic review and meta-analysis. Br J Cancer.

[B11] Dasgupta K, Di Cesar D, Ghosn J, Rajan R, Mahmud S, Rahme E (2006). Association between nonsteroidal anti-inflammatory drugs and prostate cancer occurrence. Cancer J.

[B12] Mahmud SM, Tanguay S, Begin LR, Franco EL, Aprikian AG (2006). Non-steroidal anti-inflammatory drug use and prostate cancer in a high-risk population. Eur J Cancer Prev.

[B13] Roberts RO, Jacobson DJ, Girman CJ, Rhodes T, Lieber MM, Jacobsen SJ (2002). A population-based study of daily nonsteroidal anti-inflammatory drug use and prostate cancer. Mayo Clin Proc.

[B14] Rotem R, Tzivony Y, Flescher E (2000). Contrasting effects of aspirin on prostate cancer cells: suppression of proliferation and induction of drug resistance. Prostate.

[B15] Hsu AL, Ching TT, Wang DS, Song X, Rangnekar VM, Chen CS (2000). The cyclooxygenase-2 inhibitor celecoxib induces apoptosis by blocking Akt activation in human prostate cancer cells independently of Bcl-2. J Biol Chem.

[B16] Myers C, Koki A, Pamukcu R, Wechter W, Padley RJ (2001). Proapoptotic anti-inflammatory drugs. Urology.

[B17] Sporn MB, Suh N (2002). Chemoprevention an essential approach to controlling cancer. Nat Rev Cancer.

[B18] Garcia Rodriguez LA, Gonzalez-Perez A (2005). Use of antibiotics and risk of breast cancer. Am J Epidemiol.

[B19] Kaye JA, Jick H (2005). Antibiotics and the risk of breast cancer. Epidemiology.

[B20] Velicer CM, Heckbert SR, Lampe JW, Potter JD, Robertson CA, Taplin SH (2004). Antibiotic use in relation to the risk of breast cancer. JAMA.

[B21] Dennis LK, Dawson DV (2002). Meta-analysis of measures of sexual activity and prostate cancer. Epidemiology.

[B22] Mandel JS, Schuman LM (1987). Sexual factors and prostatic cancer: results from a case-control study. J Gerontol.

[B23] Pienta KJ, Esper PS (1993). Risk factors for prostate cancer. Ann Intern Med.

[B24] Key T (1995). Risk factors for prostate cancer. Cancer Surv.

[B25] Hayes RB, Pottern LM, Strickler H, Rabkin C, Pope V, Swanson GM, Greenberg RS, Schoenberg JB, Liff J, Schwartz AG, Hoover RN, Fraumeni JF (2000). Sexual behavior, STDs and risks for prostate cancer. Brit J Cancer.

[B26] Rosenblatt KA, Wicklund KG, Stanford JL (2000). Sexual factors and the risk of prostate cancer. Amer J Epidemiol.

[B27] Fowler JE (2002). Antimicrobial therapy for bacterial and nonbacterial prostatitis. Urology.

[B28] Krieger JN, Riley DE, Vesella RL, Miner DC, Ross SO, Lange PH (2000). Bacterial DNA sequences in prostate tissue from patients with prostate cancer and chronic prostatitis. J Urology.

[B29] Hankey BF, Feuer EJ, Clegg LX, Hayes RB, Legler JM, Prorok PC, Ries LA, Merrill RM, Kaplan RS (1999). Cancer surveillance series: interpreting trends in prostate cancer – part I: Evidence of the effects of screening in recent prostate cancer incidence, mortality, and survival rates. J Natl Cancer Inst.

